# Draft Whole-Genome Sequence of OXA-48-Producing Multidrug-Resistant *Klebsiella pneumoniae* KP_ST11_OXA-48

**DOI:** 10.1128/genomeA.00737-14

**Published:** 2014-07-31

**Authors:** Jennifer Villa, Esther Viedma, Patricia Brañas, Jesús Mingorance, Fernando Chaves

**Affiliations:** a Servicio de Microbiología Clínica, Hospital Universitario 12 de Octubre, Madrid, Spain; b Spanish Network for Research in Infectious Diseases (REIPI RD12/0015), Instituto de Salud Carlos III, Madrid, Spain; c Hospital Universitario la Paz, IdiPAZ, Madrid, Spain

## Abstract

We present the draft genome sequence of a blood culture isolate of OXA-48-producing *Klebsiella pneumoniae* (sequence type 11 [ST11]) obtained in the course of a hospital outbreak in Spain. Sequence analysis showed 121 genes related to antibiotic and antiseptic resistance, including *bla*_OXA-48_, which was located in an IncL/M plasmid.

## GENOME ANNOUNCEMENT

Nosocomial pathogen *Klebsiella pneumoniae* is associated with serious illnesses, and the problem is aggravated due to its propensity to acquire multidrug resistance (1, 2). The spread of OXA-48 producers is now considered an endemic threat in *K. pneumoniae* and represents an important source of multidrug resistance in Europe (3, 4).

The *K. pneumoniae* strain KP_ST11_OXA-48 was isolated from a blood culture in the setting of a large outbreak by an OXA-48-producing multidrug-resistant *K. pneumoniae* clone affecting a total of 44 patients. The strain belonged to sequence type (ST) 11. This clone emerged in our hospital in 2013 and currently persists despite the control measures implemented. The strain showed multiple resistances to clinically used antibiotics, including all β-lactams (ertapenem, meropenem, and imipenem showed MICs of ≥32 µg/ml), fluoroquinolones, aminoglycosides (except for amikacin), sulfonamides, macrolides, and tetracyclines. It was susceptible to colistin and fosfomycin.

Next-generation sequencing was performed on a Roche 454 Junior sequencer to generate 31.0-fold coverage (2 runs). *De novo* assembly was performed using Roche Newbler version 2.7 (Roche) and generated 185,726,039 bp and 400,168 reads. The KP_ST11_OXA-48 assembly resulted in 124 contigs, with an *N*_*50*_ contig size of 203,680 nucleotides and a total length of 5,580,994 bp. Contigs were annotated using the Prokaryotic Genomes Automatic Annotation Pipeline (PGAAP) through NCBI (http://www.ncbi.nlm.nih.gov/), providing a total of 5,342 genes, 5,198 coding DNA sequence genes, 41 pseudogenes, 8 rRNAs (5S, 16S, and 23S), 81 tRNAs, and 14 noncoding RNAs (ncRNAs). Analyses of antibiotic, biocide, and metal resistance were performed with the Comprehensive Antibiotic Resistance database (CARD) and the Antibacterial Biocide and Metal Resistance Genes Database (BacMet) (5, 6).

This approach highlighted the presence of 121 genes related to antibiotic, antiseptic, and toxic compound resistance, including genes associated with specific resistance to β-lactams (*bla*_SHV-11_, *bla*_ACT_, *bla*_CTXM-15_, *bla*_OXA-1_, and *bla*_OXA-48_), aminoglycosides [*aac*(*6*')*-Ib-cr*, *aac*(*3*)*-IIa*, *aadA2*, and *aph*(*3′*)*-Ia*], fluoroquinolones (*qnrB1*), macrolides (mph-A), phenicol (*catA1* and *catB2*), and sulfonamide-trimethoprim (*sul1* and *dfrA12*), as well as resistance determinants to metals such as arsenic, copper, cobalt-zinc-cadmium, chromium, and mercury. The genome showed the presence of one integron that is composed of the gene cassette array *intI-dhfrA12-aadA2-qac*Δ*1*/*sul1*, named In 27 by the Integron Database (http://integrall.bio.ua.pt/). In addition, we observed alterations in the *gyr*A subunit and the *par*C subunit that play major roles in the development of ﬂuoroquinolone resistance. Also, we found the virulence factor P-related fimbriae regulatory gene (*prfB*) and a biocide resistance gene (*oqxA-B*) that coselect antibiotics such as chloramphenicol, ciprofloxacin, and trimethoprim and toxic compounds such as chlorhexidine, sodium dodecyl sulfate, benzylkonium chloride, and triclosan. The analysis of the genome revealed sequences that belonged to four plasmids (IncFIB, IncFII, IncL/M, and IncR groups). This strain showed that the *bla*_OXA-48_ gene was located in the IncL/M plasmid (62,811 bp), which includes a *tir* gene that is truncated by a *bla*_OXA-48_ gene.

We present the sequence of an OXA-48-producing *Klebsiella pneumoniae* isolate belonging to ST11, a clone that is often associated with hospital outbreaks and is the major clone of OXA-48 producers in the Madrid region. This sequence information might be a useful tool to study the behavior of these epidemic strains that have spread rapidly and can persist over time in health care institutions.

### Nucleotide sequence accession number.

The draft genome sequence of *Klebsiella pneumoniae* KP_ST11_OXA-48 has been included in the GenBank whole-genome shotgun (WGS) database under the accession no. JNHB00000000.
